# Comparison of dimension reduction methods on fatty acids food source study

**DOI:** 10.1038/s41598-021-97349-6

**Published:** 2021-09-21

**Authors:** Yifan Chen, Yusuke Miura, Toshihiro Sakurai, Zhen Chen, Rojeet Shrestha, Sota Kato, Emiko Okada, Shigekazu Ukawa, Takafumi Nakagawa, Koshi Nakamura, Akiko Tamakoshi, Hitoshi Chiba, Hideyuki Imai, Hiroyuki Minami, Masahiro Mizuta, Shu-Ping Hui

**Affiliations:** 1 Faculty of Health Sciences, Hokkaido University, Sapporo, 060-0808 Japan; 2 School of Medical Technology, Faculty of Health Science, Gunma Paz University, 1-7-1 Tonyamachi, Takasaki, Gunma 370-0006 Japan; 3 Patients Choice Laboratories, 7026 Corporate Dr, Indianapolis, IN 46278 USA; 4 National Institutes of Biomedical Innovation, Health and Nutrition, Tokyo, 162-8636 Japan; 5 Research Unit of Advanced Interdisciplinary Care Science, Osaka City University Graduate School of Human Life Science, Osaka, 558-8585 Japan; 6 The Hokkaido Centre for Family Medicine, Sapporo, 007-0841 Japan; 7 Graduate School of Medicine, University of the Ryukyus, Nishihara, Okinawa 903-0215 Japan; 8 Faculty of Medicine, Hokkaido University, Sapporo, 060-8638 Japan; 9 Department of Nutrition, Sapporo University of Health Sciences, Sapporo, 007-0894 Japan; 10 Faculty of Information Science and Technology, Computer Science and Information Technology Mathematical Science, Hokkaido University, Sapporo, 060-0814 Japan; 11 Information Initiative Center, Hokkaido University, Sapporo, 060-0811 Japan

**Keywords:** Biomarkers, Nutrition, Public health

## Abstract

Serum fatty acids (FAs) exist in the four lipid fractions of triglycerides (TGs), phospholipids (PLs), cholesteryl esters (CEs) and free fatty acids (FFAs). Total fatty acids (TFAs) indicate the sum of FAs in them. In this study, four statistical analysis methods, which are independent component analysis (ICA), factor analysis, common principal component analysis (CPCA) and principal component analysis (PCA), were conducted to uncover food sources of FAs among the four lipid fractions (CE, FFA, and TG + PL). Among the methods, ICA provided the most suggestive results. To distinguish the animal fat intake from endogenous fatty acids, FFA variables in ICA and factor analysis were studied. ICA provided more distinct suggestions of FA food sources (endogenous, plant oil intake, animal fat intake, and fish oil intake) than factor analysis. Moreover, ICA was discovered as a new approach to distinguish animal FAs from endogenous FAs, which will have an impact on epidemiological studies. In addition, the correlation coefficients between a published dataset of food FA compositions and the loading values obtained in the present ICA study suggested specific foods as serum FA sources. In conclusion, we found that ICA is a useful tool to uncover food sources of serum FAs.

## Introduction

There is a worldwide growing attention to dietary fatty acid (FA) intake because FA metabolism is related with various health problems of heart, liver, kidney, brain, immune system and possibly of all organs^1–6^. For prevention and management of these problems, comprehensive knowledge of dietary FA uptake in an individual or a population could be useful. Quantitative FA profiling in plasma (or serum) may give the best information for this purpose. However, there is still some difficulty to interpret the dietary source of FAs in plasma.

The difficulty is related with the structural and metabolic complexities of FAs. FAs are derived from foods or synthesized de novo mainly by the liver. Linoleic and α-linolenic acids, which are classified as polyunsaturated fatty acids (PUFAs) cannot be synthesized de novo and need to be taken with foods, thus they are called as essential fatty acids (FAs). Unsaturated FAs are classified mostly into ω-3, ω-6, and ω-9 groups, which implies the position of double bond is on the third, sixth, and ninth carbon from the methyl end, while a minor portion of FAs is classified into ω-7^7^. The major three ω groups, ω-3, ω-6, and ω-9, are different from each other in dietary sources, metabolic pathways, and biological behaviors^8^. Long saturated FAs (C ≥ 12) and monounsaturated FAs can be derived from dietary sources or synthesized de novo. Some polyunsaturated FAs, such as eicosapentaenoic acid (EPA) and docosahexaenoic acid (DHA), need to be obtained from food^7,9,10^. Thus, plasma FAs reflect intestinal uptake, hepatic biosynthesis, and additionally adipose lipolysis. To be more complicated, plasma FAs reflect both a short-term dietary intake and a stable long-term dietary intake^11^.

Moreover, in blood, FAs are mainly transported in the esterified forms, such as triglycerides (TGs), phospholipids (PLs), and cholesteryl esters (CEs). TGs occupy the largest part (98.6%) of plasma acylglycerols that include diacylglcerols (DGs) and monoacylglycerols (MGs) as minor components^12^. A minor part of plasma FAs are unesterified and mainly associated with albumin, thus called as free fatty acids (FFAs). FFAs are rapid in clearance from plasma. On the other hand, esterified FAs reside in plasma lipoproteins, namely, chylomicrons, very-low-density lipoproteins (VLDLs), low-density lipoproteins (LDLs), and high-density lipoproteins (HDLs). These lipoproteins have distinctive metabolic rates and pathways that can be fluctuated by various factors including physical activity, nutrition, and metabolic conditions. Thus, plasma FAs reflect the complexity of lipoprotein metabolism in circulation.

There are previous studies about FA and its sources. The association of FAs contained in plasma lipid fractions (TG, PL, CE, and FFA) with food frequency questionnaire (FFQ) confirmed by Spearman’s correlation coefficients was reported previously^13–15^. In another study for various Swedish populations, FAs in plasma PL and CE fractions were considered as interchangeable biomarkers for dietary fat intake^16^. The combination of FAs in plasma TG and FFA fractions was reported to generate a good correlation with dietary FA intake^17^. However, it is clear that the association of plasma FAs with dietary consumption should be automatically limited to the FAs that are not endogenously synthesized^11^. Hence, plasma FAs are regarded as low specific biomarkers^18^. Statistical analysis methods to extract specific information from complicated FA datasets are strongly desired.

Some statistical analysis methods, such as regression analysis and t-tests, have been used in previous FA concerning reports^19–25^. Nevertheless, only limited information of the use of dimension reduction methods in a large-scale human serum study is available, while principal component analysis (PCA) and factor analysis were reported^26^. In this report, we compared the usefulness of four dimension reduction methods, including independent component analysis (ICA), common principal component analysis (CPCA), factor analysis, and PCA in an epidemiological study in Japan. ICA, in particular, is known to generate independent variables. “Independence” is a stricter concept than “uncorrelation” generated by PCA, CPCA, and factor analysis. ICA may provide better results than other methods in differentiation of FA sources.

In our present study, on the basis of the serum concentrations of CEs, FFAs, and TFAs measured, we calculated the FA concentrations in TG + PL fraction. Then we combined the data of all lipid fractions (CE, FFA, and TG + PL) into a single dataset and studied the relationship between FAs and dietary sources.

## Results

### Results from ICA

**Figure 1:**
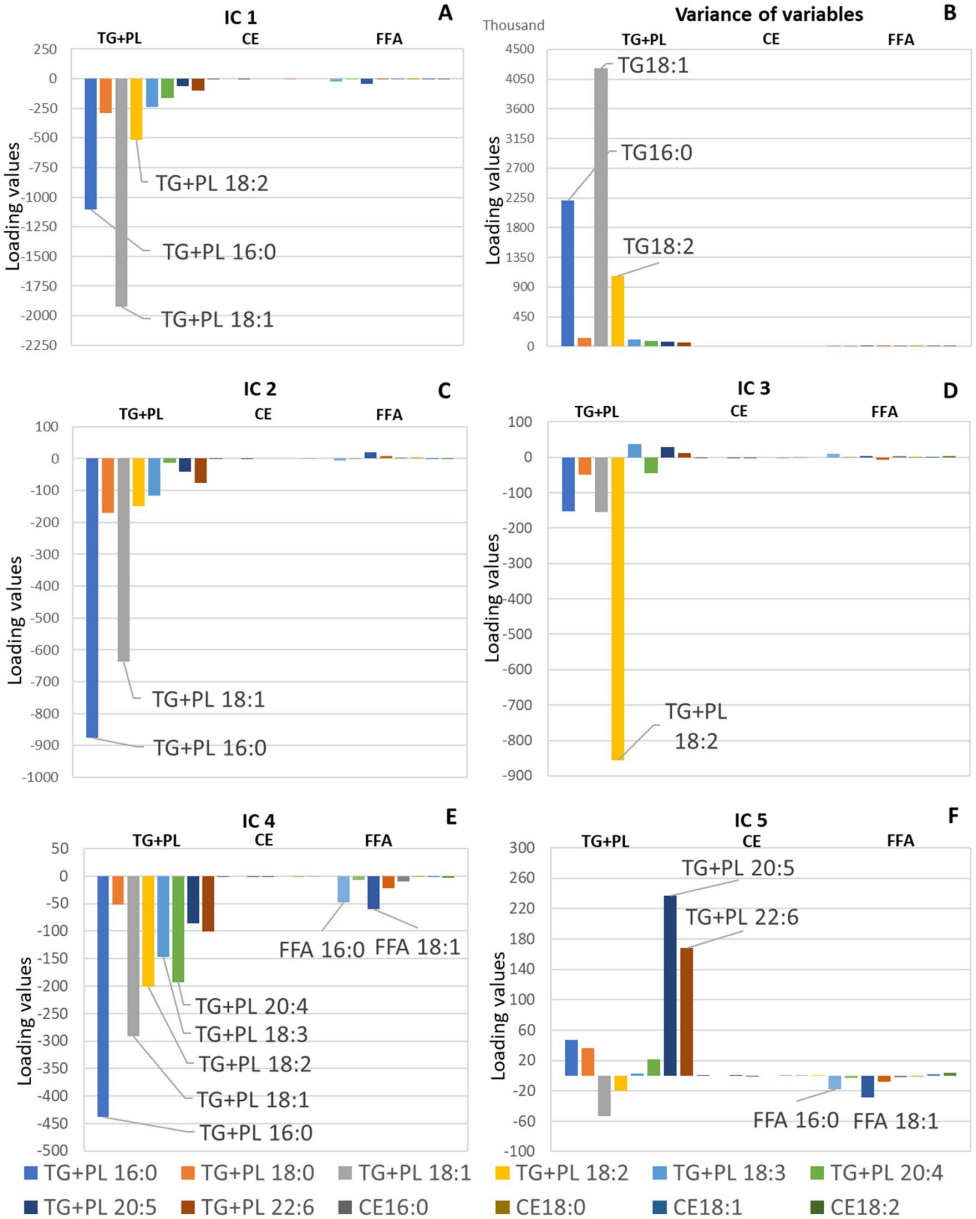
Histograms of independent component analysis results, including (**A**) IC 1, (**B**) variance of variables, (**C**) IC 2, (**D**) IC 3, (**E**) IC 4, and (**F**) IC 5. The omega groups of FAs were omitted.

In the results of ICA, outcomes of ICA model with five components generated the outcomes meet our expectation best. As to the first independent component (IC 1) (Fig. 1A), the histogram displayed that variables with high loading values were TG + PL 18:1n-9 and TG + PL 16:0, which are commonly with highest concentration in plasma. Additionally, the variation value of each subtype is in the same order as the value of IC 1 (Fig. 1B), thus IC 1 was regarded as size factor.

The histogram of the second independent component (IC 2) displayed that variables with high loading values were TG + PL 16:0 and TG + PL 18:1n-9 (Fig. 1C), which can be obtained from animal fats and synthesized in human body. Since, the loading values of FFAs, which are influenced either by dietary intake or adipose tissues lipolysis^8,27^, are extremely low in IC 2, thus we regarded the IC 2 as representation of the endogenous fatty acids.

The histogram of the third independent component (IC 3) (Fig. 1D) displayed that TG + PL 18:2n-6, which cannot be synthesized in human body, was with highest loading value. Dietary sources of FA 18:2n-6 are generally vegetables, nuts, and seeds. Moreover, we found that other essential FAs, which are obtained from meat, were with low loading values. Therefore, it is reasonable to consider IC 3 as the representation of plant oil intake^28,29^.

In the histogram of forth independent component (IC 4) (Fig. 1E), we found that the loading values of FFA 16:0 and FFA 18:1n-9 were also relatively high compared with those in first three independent components. This situation informed us that IC 4 could the biomarker to reflect exogenous FAs, because FFAs are known to reflect food intake during fasting. More precisely, the main sources of serum FFAs are food intake, liver synthesis and adipose lipolysis during fasting^8^. Nevertheless, mobilization of serum FFA from adipose tissues was reported to be selective, thus, we thought that serum FFA variables only serves as a biomarker to mark the exogenous FAs^17,27^. In addition, the histogram of IC4 also displayed that TG + PLs 16:0, 18:1n-9, 18:2n-6, 20:4n-6 and 18:3 (omega group was not given because of the presence of 18:3n-3 and 18:3n-6) were with relatively high loading values. TG + PL 16:0 and TG + PL 18:1n-9 can be synthesized in human body as well as obtained from food intakes. TG + PLs 18:2n-6, 18:3, and 20:4n-6 are regarded as essential FAs, which need to be gained from food intakes. The common point of those FAs source is their exogenous source, which confirmed us that TG + PL variables in IC 4 can reflect the exogenous FAs. To specify the sources of exogenous FAs, we calculated the correlation coefficients between loading values of TG + PL variables and reported adipose FA composition for Japanese, which is low ($\rho =0.61$)^30^. Therefore, we refused the assumption that IC 4 reflects the adipose composition but accepted the suppose that IC 4 represents exogenous FAs. According to the correlation coefficients between loading values of TG + PL variables and published food FA concentration, we deemed the major exogenous FA sources is animal meat fat^29^. The details concerning exact food sources will be described in the discussion part of this paper.

The histogram of fifth independent component (IC 5) presented that TG + PL 20:5n-3 and TG + PL 22:6n-3 were with high loading values (Fig. 1D). TG + PL 20:5n-3 and TG + PL 22:6n-3 are eicosapentaenoic acid (EPA) and docosahexaenoic acid (DHA), which are derived from fish oil. Furthermore, FFA 16:0 and FFA 18:1n-9, influenced by food intake and adipose tissues lipolysis, were with relatively high loading values compared with the first three components. Therefore, the IC 5 was judged to represent fish oil intake.

### Results from factor analysis

**Figure 2:**
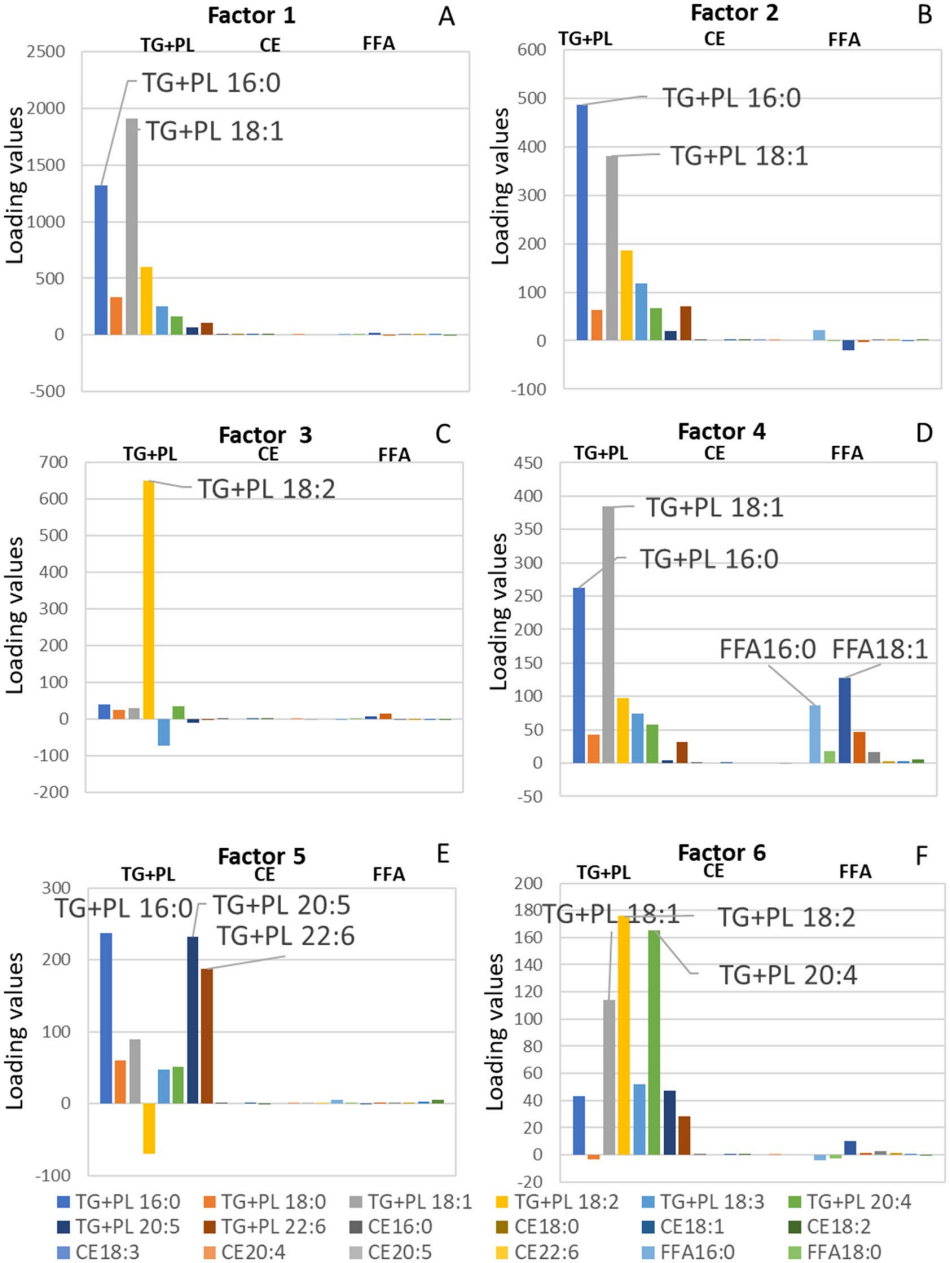
Histograms of factor analysis results, including (**A**) Factor 1, (**B**) Factor 2, (**C**) Factor 3, (**D**) Factor 4, (**E**) Factor 5, and (**F**) Factor 6.

In the result of factor analysis, the model with 6 factors generated the best result. Therefore, we only explain the model generating 6 factors. The histogram of first factor (Factor 1) displayed that variables with high loading values were TG + PL 18:1n-9 and TG + PL 16:0 (Fig. 2A), which are the variables with highest concentration in serum. Therefore, Factor 1 was considered as the size factor for the same reason as we explained in the description (Fig. 1B).

The histogram of the second factor (Factor 2) displayed that variables with high loading values were TG + PL 16:0 and TG + PL 18:1n-9 (Fig. 2B), which can be synthesized in human body. Besides, the loading values of FFA was in low level. Therefore, Factor 2 was judged to represent endogenous FAs.

The histogram of the third factor (Factor 3) presented that TG + PL 18:2n-6 was with extremely high loading value (Fig. 2C). Thus, Factor 3 was deemed to represent plant oil intake for the same reason in the former sections.

The histogram of the fourth factor (Factor 4) displayed that variables with high loading values were TG + PL 16:0 and TG + PL 18:1n-9 (Fig. 2D). In addition, the loading values of FFA 18:1n-9 and FFA 16:0 were also comparatively high, therefore Factor 4 was considered to reflect the animal fat intake with the same reason in the description of ICA results.

The histogram of the fifth factor (Factor 5) exhibited that variables with high loading values were TG + PLs 16:0, 20:5n-3 and 22:6n-3 (Fig. 2E). TG + PL 20:5n-3 and TG + PL 22:6n-3 are majorly obtained from fish oil intake, thus Factor 5 was considered to represent fish oil intake.

The histogram of the sixth factor (Factor 6) displayed that variables with high loading values were TG + PLs 18:2, 20:4n-6 and 18:1n-9, which are in ω-6 group (Fig. 2F). TG + PL 20:4n-6 can be generated from TG + PL 18:2n-6 after elongation and desaturation synthesis. Thus, Factor 6 was claimed to represent ω-6 group metabolism.

### Results from CPCA

**Figure 3:**
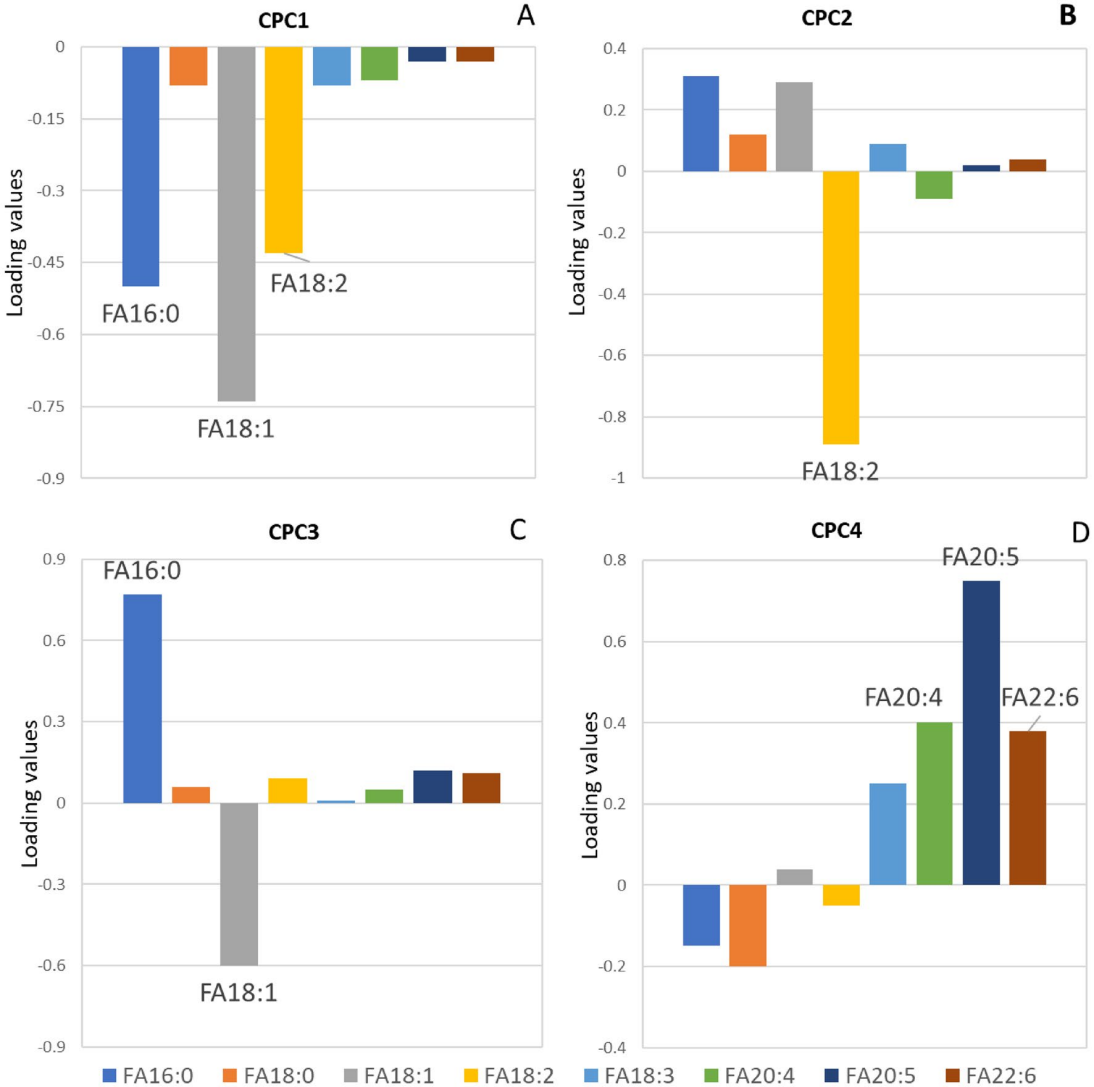
Histograms of CPCA results, including (**A**) CPC1, (**B**) CPC2, (**C**) CPC3, and (**D**) CPC4.

In CPCA, TG + PL, FFA, and CE were combined as one set, which was denoted as FA. The histogram of the first common principal component (CPC1) displayed that variables with high values were FAs 18:1n-9, 16:0 and 18:2n-6, whose concentration is commonly high in serum (Fig. 3A). It is considered as size factor for the same reason in the explanation of IC1 (Fig. 1B).

The histogram of the second common principal component (CPC2) displayed that variable with high value was FA 18:2n-6 (Fig. 3B). On the contrary, FA 16:0 and FA 18:1n-9 were with low loading values in CPC2. Therefore, CPC2 was judged as the representation of plant oil intake.

The histogram of the third common principal component (CPC3) displayed that variables with high values were FA16:0 and FA18:1n-9 (Fig. 3C). Furthermore, the arrows of FA 16:0 and FA 18:1n-9 were in converse directions, which might indicate the inverse relationship between the two FAs in the inner synthesis process^31^. Besides, the concentration of serum FA 16:0 and FA 18:0 in common diet group was found similar to those in vegan group^32^. This situation is caused by the self-producing ability of non-essential FAs in our body. Thus, it is hard for us to judge the FA 16:0 and FA18:0s’ sources with CPCA. We supposed CPC3 could be either the representation of inner non-essential FAs metabolisms or the representation of meat fat intake.

As to the fourth common principal component (CPC4) (Fig. 3D), the CPC4 axis displayed that variables with high values were FAs 20:5n-3, 20:4n-6 and 22:6n-3. Concentration of FA 20:5n-3 and FA 22:6n-3 are high in fish oils, and FA 20:4n-6 can be partly gained from fish^33^. Therefore, CPC4 was deemed to represent fish oil intake.

There are other four common principal components in our study, but the loading values of them are unclear. Thus, we do not explain the representation of the rest common principal components.

### Results from PCA

**Figure 4:**
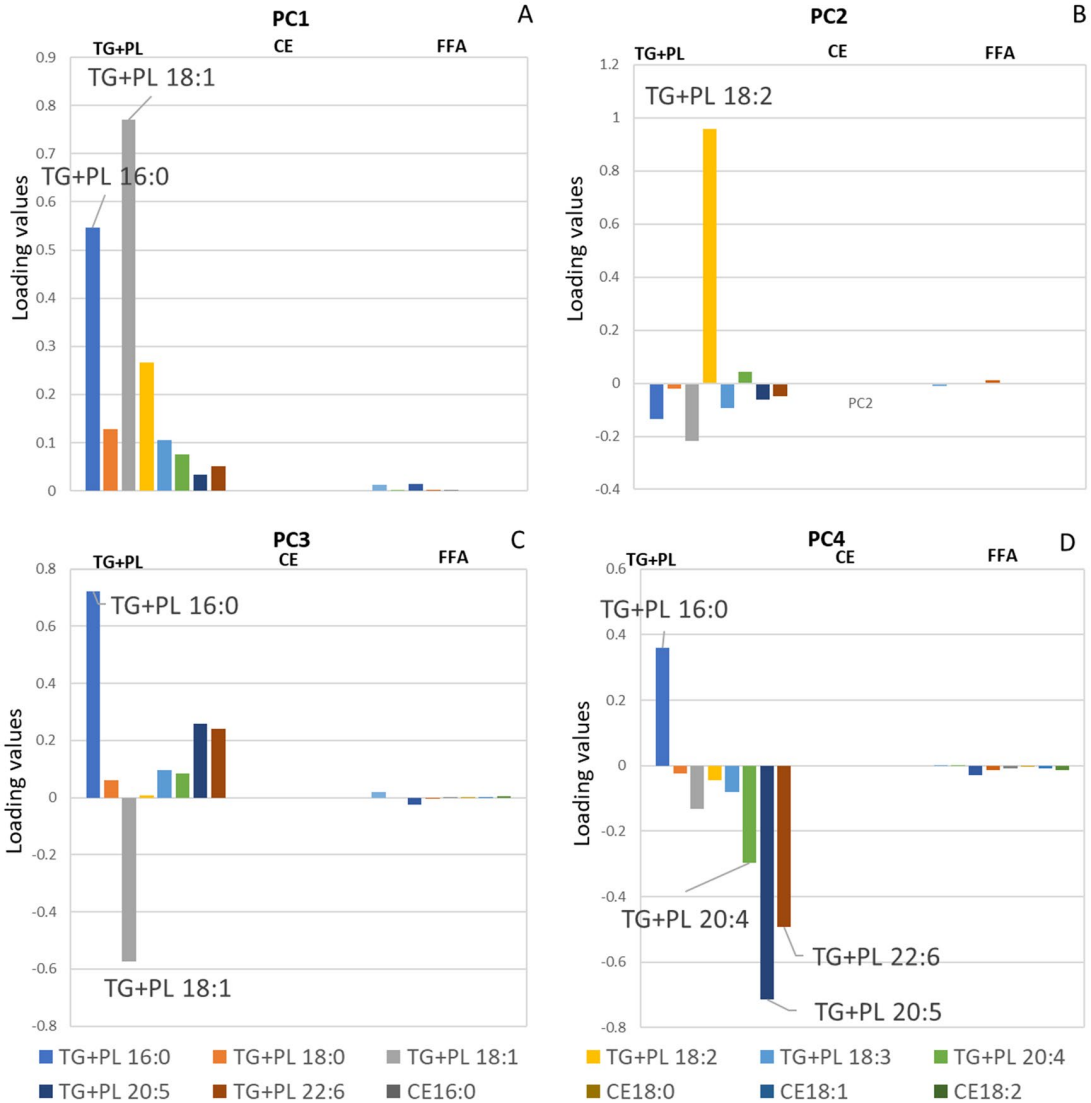
Histograms of PCA results, including (**A**) PC1, (**B**) PC2, (**C**) PC3, and (**D**) PC4.

In the results of PCA, the histogram of first principal component (PC1) showed that variables with high loading values were TG + PL 18:1n-9 and TG + PL 16:0 (Fig. 4A). Therefore, it was considered as size factor for the same reason in the explanation of ICA. The histogram of the second principal component (PC2) displayed that variables with high loading values were TG + PL 18:2n-6, thus PC2 was considered as plant oil intake.

As to the third principal component (PC3), the histogram (Fig. 4B) displayed that TG + PL 16:0 and TG + PL 18:1n-9 were with high loading values but in converse directions. We thought PC3 probably reflect the metabolism of TG + PL 16:0 and TG + PL 18:1n-9. However, TG + PL 20:5n-3 and TG + PL 22:6n-3 were with relatively high loading values in PC3, thus we found it is difficult to explain PC3.

The histogram of the fourth principal component (PC4) exhibited that variables with high loading values were TG + PLs 20:5n-3, 22:6n-3, 16:0 and 20:4n-6 (Fig. 4C). Thus, PC4 was considered to represent fish oil intake. We do not explain principal components after PC4 in this study because they were considered obscure (Fig. 4D).

### Results of correlation coefficients between food FA concentration and loading values in ICA

**Figure 5:**
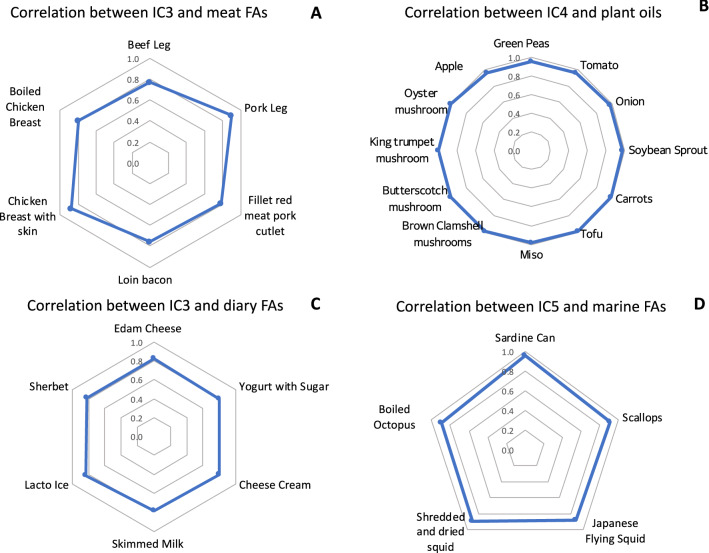
Diagram of suggested food sources. We calculated the correlation coefficients (CCs) between the loading values in the ICA results and the published FA composition in foods^29^. Then, we selected the foods with the top 10 highest CCs in four major food sources of animal meats (**A**), plants (**B**), dairy products (**C**), and marine foods (**D**).

In addition, we specified the food sources by correlation coefficient (CC) between the loading values of IC 3, IC 4, and IC 5 and the standard tables of food composition in Japan, 2015^29^. In this process, the foods with the top 10 highest CC values in four major food groups, namely, animal foods, dairy products, marine foods, and plants, were selected as the potential food sources (Supplementary). For animal foods, animal meats and dairy products were studied separately (Fig. 5A,C). For plant FA sources, fruits, mushrooms, vegetables and beans (including Tofu and Miso) were studied (Fig. 5B).

According to the CC values, the main food sources of animal meats were suggested as pork leg, chicken breast with skin, boiled chicken breast, meat pork cutlet, beef leg, and loin bacon (Fig. 5A). As to the plant oil sources, the main food sources were suggested as tofu, mushrooms, carrot, onion, and so on (Fig. 5B). As to the food sources of dairy FAs, the main food sources were suggested as cheeses, yogurt, skimmed milk, sherbet and Lacto ice (a common Japanese ice-cream containing milk-solids content of 3% or greater) (Fig. 5C). As to the marine food sources, the main food sources were suggested as sardines, squids, scallops, and octopus (Fig. 5D), although salmon, tuna, or other commonly eaten fishes in Japan were not suggested. It is because sardines serve as forage food for other fishes^34^, and because sardines are the most common fish in the fishery industry, as it accounted for 23% of the total fish capture amount in 2015, Japan^35^.

## Discussion

Dietary FA source is generally evaluated by food frequency questionnaires (FFQs). However, FFQs heavily depend on accuracy in self-report and reliability of the food databases^17^. Thus, it is crucial to explore objective and unbiased methods in addition to FFQ. In this study, four methods (ICA, CPCA, factor analysis and PCA) were compared in performance of suggesting food sources of serum FAs. ICA generated the most suggestive results: IC1 for the size factor; IC2 for endogenous synthesis; IC3 for plant oil intake; IC4 for animal fat intake; and IC5 for fish oil intake (Fig. 1). Factor analysis was also found useful (Fig. 2). The Factor 6 was considered to represent the ω-6 FAs (Fig. 2F). Since the ω-6 FAs distribute among various food sources including animal meats, fish and plants, the Factor 6 seemed to represent the structural property of FAs rather than food sources. Thus, we recommend ICA rather than factor analysis for better exploration of dietary sources of serum FAs. The different results between factor analysis and ICA was originated from the different statistic consideration. Factor analysis generates uncorrelated factors, while ICA generates independent components^36^.

As described in the introduction, “independence” is stronger than “uncorrelation”, because uncorrelation reflects only linear independence but not non-linear independence. On the other hand, independence reflects the both^36^. Non-linear relationship has been reported for FA and PL metabolisms in previous studies^37,38^. In another study, the relationship between plasma and brain DHA levels was suggested to be nonlinear^38^. Besides, total plasma concentration of ω-3 FAs and ω-6 FAs were claimed to be in a non-linear relationship between fat mass, which should have an influence on plasma free FAs^39^. In conclusion, both linear and non-linear correlation exist in plasma FA metabolism. Therefore, dimension reduction methods only diminish linear correlation, such as factor analysis and PCA, are not enough for our study. In addition, the metabolism of FAs is affected by various factors such as food sources, appetite, physical activities, diurnal rhythm changes^16^, as well as intestinal microbiota, intestinal absorption, lipoprotein metabolism, and oxidative conditions^40^.

In this study, we studied CCs between the FA concentration chart of food and the loading values in factor analysis as well. However, the results were unconvincing, for beef was suggested to be the main source among animal meats, and shellfish was suggested as the main source among marine foods (Supplementary Fig. S1). These situations are unlikely to happen in Japan. Because the Japanese domestic consumption of beef, pork, and chicken were 19.6%, 41.5%, and 38.1%, respectively in 2015, and the domestic fish production of fish, shellfish and squid were 66.7%, 15.5%, and 3.6% respectively in 2015^35,41^. Thus, factor analysis was shown not powerful in separating independent “signal” sources, namely food sources in this study, according to the previous report^36^.

According to the histograms (Figs. 1, 2, 3, 4), CPCA and PCA cannot compete in accuracy with ICA and factor analysis. Firstly, the components of PCA and CPCA didn’t reflect the FA dietary sources well, while ICA and factor analysis generated more suggestive results. Secondly, CPCA and PCA cannot distinguish endogenous and exogenous FAs. Based on the comparisons in this study, factor analysis, CPCA, and PCA are not recommended as the choice of method for exploring the food sources of serum FAs.

It has been difficult to differentiate animal fat intakes and human endogenous FAs because their compositions are similar^18^. Endogenous FAs include saturated and monounsaturated FAs, which is similar with animal fat FA composition. The de novo lipogenesis (DNL) index (FA16:0/FA18:2n-6) was reported to reflect the endogenous FAs in the high carbohydrate dietary intake groups^42^. However, the DNL index is hardly practical for normal dietary groups, which take considerable amounts of animal fat. Therefore, ICA could serve as a valuable method for detecting the food sources of serum FAs.

Regarding the dataset used in this study, it contained a combined FA data including FFA, CE, and TG + PL. Combined FA datasets have been studied in previous reports^17,43^. A combined dataset is better than a dataset of a single lipid fraction, because the latter ignores the dynamism of lipid metabolism as follows. Firstly, the structure of a plasma lipid fraction varies to that of another lipid fraction under physiological regulations. For example, TG can be hydrolyzed to release FFAs, and vice versa. Thus, FA is rapidly exchanged among lipid fractions. Therefore, FAs in a single lipid fraction are not reflective enough of dietary uptake. Secondly, plasma FFAs during fasting are originated from adipose tissues and reflect the composition of adipose fat^44^, although FFA mobilization from adipose tissues is also selective^27^. Finally, plasma TG and CE reflect short-term dietary intake, while plasma PL reflects long-term diet^17^. In the present study, we combined the different plasma lipid fractions to avoid the bias and limitations based on the above conditions.

There are limitations in this study, however, that we obtained PL + TG variables by substruction of FFA and CE from Total FA instead of the exact measurement. Thus, in the future, exact concentrations of PL and TG should be measured by molecule-specific mass spectrometry for each lipid fraction. Besides, the ICA algorithm used in this study was the most basic version that we selected for convenience^36^. A more advanced ICA algorithm should be studied in future.

## Conclusions

There are two major findings in this study. Firstly, a dataset consists of different lipid fractions is superior to a dataset of single lipid fraction, reflecting the dynamic lipid metabolism. Secondly, ICA is suggested to be more useful in detecting dietary sources of serum FAs and in differentiation between exogenous and endogenous FAs compared with factor analysis, CPCA, and PCA. This merit of ICA could be an advantage in discovery of potential biomarkers and might possibly complement FFQ. In conclusion, bioinformatic approach is beneficial to obtain valuable suggestions in epidemiological studies on serum FAs.

## Methods

### Blood samples of free fatty acids (FFAs), total fatty acids (TFAs), and cholesterol esters (CEs)

The present study was a cross-sectional study conducted as a work of the Dynamics of Lifestyle and Neighborhood Community on Health Study (DOSANCO Health Study). Briefly, the DOSANCO Health Study was a community-based study conducted in Suttu town, Hokkaido, Japan, during the year of 2015^45^. A total of 2100 participants of approximate 3100 population (977 men and 1123 women; 79.6% of all residents aged 3 years or more other than those living at nursing homes) completed a self-administered questionnaire. Of the 2100 participants, 1379 participants between the ages of 35 and 79 years were additionally asked to provide blood samples, and 545 participants (245 men and 300 women) complied^45^.

The study protocol was approved by the ethics committees of the Faculty of Medicine (15-002, 16-007) and the Faculty of Health Sciences (16-10), Hokkaido University. Written informed consent was obtained from all participants. The study was carried out in accordance with the Declaration of Helsinki (World Medical Association).

Blood was drawn after an overnight fast. After blood coagulation at room temperature, serum was separated by centrifugation at 4 °C and stored at − 80 °C for no longer than 3 years before analysis. The samples were confirmed to be stable at this condition.

### Measurements of FFAs, TFAs and CEs

Free and total FAs, and CEs were measured by the methods described earlier^46–50^. Briefly, serum FFAs and TFAs were determined by acyl-specific High Performance Liquid Chromatography (HPLC) with labeling as previously described. Serum CEs were determined by acyl-specific Liquid Chromatography with tandem mass spectrometry (LC–MS/MS). The details of the quantitative methods are provided in Supplementary material. The methods were validated in accordance with the Clinical and Laboratory Standards Institute guideline^51^.

### Datasets of free and total fatty acid

Since fatty acid in the serum can exist in both esterified, we applied clinical multivariate analysis to both total and free fatty acid datasets. The dataset consists of the subtypes of 16 TFA: TFA 4:0, TFA 6:0, TFA 12:0, TFA 14:0, TFA16:0, TFA18:0, TFA18:1n-9, TFA18:2n-6, TFA 18:3, TFA 20:0, TFA 20:4n-6, TFA 20:5n-3, TFA22:6n-3, TFA22:0, TFA24:0, and TFA26:0. FFA dataset consists of the subtypes of 12 FFA: FFA4:0, FFA6:0, FFA12:0, FFA14:0, FFA16:0, FFA18:0, FFA18:1n-9, FFA18:2n-6, FFA18:3, FFA20:4n-6, FFA20:5n-3, and FFA 22:6n-3. The latter dataset lacks FFA20:0, FFA22:0, FFA24:0, and FFA26:0, because they were not detected.

TFAs and FFAs are affected by diet, and therefore, they are potential confounders. However, since the information on diet was not available in this study, we didn't discuss these confounding variables.

### Datasets of cholesterol esters

The CE dataset contained 8 subtypes of CE molecules: cholesteryl palmitate (CE16:0), cholesteryl stearate (CE18:0), cholesteryl oleate (CE18:1n-9), cholesteryl linoleate (CE18:2n-6), cholesteryl linolenate (CE18:3), cholesteryl arachidonate (CE20:4n-6), cholesteryl eicosapentaenoate (CE20:5n-3) and cholesteryl docosahexaenoate (CE22:6n-3).

### The combined datasets

In this study, we combined three groups of fatty acid subtypes into a new dataset with 24 subtypes: FFA 16:0, FFA 18:0, FFA 18:1n-9, FFA 18:2n-6, FFA 18:3, FFA 20:4n-6, FFA 20:5n-3, FFA 22:6n-3, TG + PL 16:0, TG + PL 18:0, TG + PL 18:1n-9, TG + PL 18:2n-6, TG + PL 18:3, TG + PL 20:4n-6, TG + PL 20:5n-3, TG + PL 22:6n-3, CE 16:0, CE 18:0, CE 18:1n-9, CE 18:2n-6, CE 18:3, CE 20:4n-6, CE 20:5n-3, and CE 22:6n-3. In this combined dataset, we aim to remove the influence of FFA and CE from TFA, thus we subtracted CE and FFA from TFA to generate TG + PL subtypes correspondingly. It can be represented below:

1 $${\text{TG}} + {\text{PL}} = {\text{TFA}} - {\text{FFA}} - {\text{CE}},$$

where TG and PL means FA contained in triglyceride and phospholipid, respectively. In Eq. (1), the concentrations were expressed as a molar concentration. TG represents the most part (> 98%) of acylglycerols in serum, whereas DG (diacylglycerols) and MG (monoacylglycerols) are negligible (< 2%)^12^. Therefore, there are reasonable grounds for approximating TG + PL using the formula of [TFA-FFA-CE].

Besides, as for the application of CPCA, we denoted every subtypes in the results as FA 16:0, FA 18:0, FA 18:1n-9, FA 18:2n-6, FA 18:3, FA 20:4n-6, FA 20:5n-3 and FA 22:6n-3. FA represents the admixture of free fatty acid, total fatty acid and cholesteryl ester.

### Statistical analyses

In this research, dimension reduction methods, including ICA, factor analysis, CPCA and PCA, were applied to the serum FA dataset which contains eight FFA, eight CE, and eight TG + PL variables. Statistical calculation was conducted by R software (https://cran.rstudio.com/), and figures were produced by Microsoft Excel.

### ICA

ICA is a basic dimension reduction method generally applied in researches of signal. ICA is manipulated to separate the mixed signal into independent subcomponents. This process is based on two principals: minimizing mutual information and maximizing non-Gausainity^52^. ICA generates independent components, while PCA and factor analysis generate uncorrelated components. From the view of statistical analysis, independence is stricter than uncorrelation, since uncorrelation equals linear independence but cannot account for the nonlinear occasion.

In this research, “ICAimax” algorithm in R software was conducted, which is fabricated to find the orthogonal rotation matrix by maximizing the joint entropy of a nonlinear function of the estimated sources. Once the orthogonal rotation process conducted, independent component can be generated by maximizing their non-Gaussianity through maximizing their kurtosis.

### CPCA

CPCA is a developed dimension reduction method from classic PCA. In CPCA, we assume there are K groups of samples, and our aim is to find out the common characteristics among the K groups. it is stated to be useful to transform the data of all K groups simultaneously to common principal components (CPCs) with large enough variances (important enough features) and to discard the CPCs with relatively small variances (unimportant features).

In our study, we set K as 3 to represent three groups of fatty acids: TG + PLs, FFAs and CEs. Dimension p was set to be 24 representing corresponding 8 subtypes of total fatty acids, free fatty acids and cholesteryl esters. The dimension r of new dataset turned out to be 8. Our goal is to find out the common vectors representing the common characteristics of the three groups of fatty acids. The package “cpca” in R software was applied to the merged dataset of three groups of FAs^53,54^.

### Factor analysis

Factor analysis is a basic dimension reduction method and well-known in psychology study. It is originated to explain latent factors behind observed phenomenon (dataset). However, being different from PCA, factor analysis is under the postulation that error exists in each variable. Hence, we may quantify the unique characteristic of each variable derived from the error, which is called “uniqueness” in factor analysis.

Mostly, two types of the rotation method are provided in factor analysis: one is orthogonal rotation method, the other is oblique rotation method. Orthogonal method is recommended under the assumption that variables are uncorrelated, whereas oblique method is recommended under the assumption that variables are correlated. In this research, the “fa” function, with orthogonal rotation method, in R software was conducted to transform the observed data into factors in lower dimension.

### PCA

PCA is one of the most basic and well-known dimension reduction methods. The basic consideration of PCA is to transform the observed dataset into a lower dimension dataset, where new variables are uncorrelated with each other. In this method, the rotation method is based on the orthogonal transformation by eigenvalues maximizing the covariance or correlation matrix of the observation.

The main aim of PCA is to reveal major information from high dimension multivariate observed data and to expose the chief factors signifying the correlation between variables. The “procomp” function in R software was conducted in this research.

## Supplementary Information

Supplementary Information.

## Data Availability

The data in this study is based on a work of the Dynamics of Lifestyle and Neighborhood Community on Health Study (DOSANCO Health Study, http://publichealth.med.hokudai.ac.jp/research/admin/). Data information is unavailable at this time, because other analyses are under study.
