# Supplementary material for: Comparison of dimension reduction methods on fatty acids food source study
Source: Sci Rep. 2021 Sep 21;11:18748. doi: 10.1038/s41598-021-97349-6 (PMC8455623; doi:10.1038/s41598-021-97349-6)
Supplement: Supplementary file 1 — Supplementary Information. [file 41598_2021_97349_MOESM1_ESM.pdf]

Supplementary

Comparison of dimension reduction methods on fatty acids food source study

Yifan Chen, Yusuke Miura, Toshihiro Sakurai, Zhen Chen, Rojeet Shrestha, Sota Kato, Emiko Okada, Shigekazu Ukawa, Takafumi Nakagawa, Koshi Nakamura, Akiko Tamakoshi, Hitoshi Chiba, Hideyuki Imai, Hiroyuki Minami, Masahiro Mizuta, and Shu-Ping Hui

Pretreatment and Measurement of free fatty acids (FFAs) and total fatty acid (TFAs)

Serum fatty acids were determined by HPLC as previously described [1,2] . Briefly, for the measurement of total fatty acids, 25 μL of serum was mixed with internal standards (IS) and saponified using KOH (0.3 M in ethanol) for 30 min at 80 °C to convert the esterified FA into free form. FAs are then labeled with 2-nitrophenylhydrazine hydrochloride (2-NPH), followed by its extraction in the hexane. The extract was completely dried under vacuum and reconstituted in methanol, filtered using a centrifugal filtering device, and injected into HPLC. For the determination of FFA, the serum sample were directly processed for derivatization without saponification[3]. Therefore, serum FA present in free from are directly labeled with 2-nitrophenylhydrazine. Chromatographic analyses were performed with a Shimadzu Nexera X2 LC-30AD UHPLC (Shimadzu Seisakusho, Kyoto, Japan) equipped with a Shimadzu Nexera X2 SIL-30AC autosampler, CTO-20A Prominence column oven and SPD-M20A photodiode array detector. C_4 Mightysil reversed-phase column (150 mm × 4.6 mm i.d., particle size 5 μm, Cica Reagent, Kanto Chemical Co., Inc, Tokyo, Japan), maintained at 35 °C was used for the separation. Gradient elution was performed with water (pH adjusted to 4.0 with trifluoroacetic acid, solvent A) and methanol (solvent B) at a flow rate of 1.0 mL/min. The absorbance of 2-NPH-labled FA was measured at 400 nm. The absolute concentration of each species of Free and total FA (μmol/L in serum) was calculated using peak area ratio to corresponding IS against the calibration curve. Concentration of free and total FA were used for multivariate analysis [4].

Sample pretreatment of cholesterol esters (CEs)

Serum (20 μL) was mixed with ethanol (200 μL) containing a mixture of ISs (1.2 nmol each) and added with hexane (1200 μL) and distilled water (1000 μL). After centrifugation at 1500×g for 10 min, the organic layer was collected and dried under vacuum (TOMY centrifugal concentrator, Tokyo, Japan). Then, the residue was dissolved in isopropanol (300 μL) and filtered using a centrifugal filtering device (PVDF 0.1 μm; Merck Millipore Ltd., Carrigtwohill, Ireland).

Finally, the sample was then diluted 8-fold with isopropanol, and 5μL was injected to LC-MS/MS[5].

Chemicals and reagents for measurement of CEs

Cholesterol and all LC/MS grade solvents including methanol, 2-propanol, n-hexane, and water purchased from FUJIFILM Wako Pure Chemical Corporation (Osaka, Japan). Ammonium acetate was purchased from Sigma-Aldrich (St. Louis, MO, USA). Other chemicals and reagents were purchased from Kanto Chemical Industry (Tokyo, Japan). Palmitic acid (FA16:0), stearic acid (FA18:0), oleic acid (FA18:1), linoleic acid (FA18:2), linolenic acid (FA18:3), and 1-(3-dimethylaminopropyl)-3-ethylcarbodiimide were purchased from Tokyo Chemical Industry Co., Ltd. (Tokyo, Japan). Arachidonic acid (FA20:4) and docosahexaenoic acid (FA22:6) were purchased from Sigma-Aldrich Co., LLC. (MO, USA). Both CE and $^{2}H_{3}$-CE (IS) were chemically synthesized as described previously and were stored stable at −80 °C for at least 3 years [6].

LC-MS/MS for CEs

The detailed conditions of LC-MS/MS for CE species and the results of validation studies were reported previously [5].

A supplementary figure

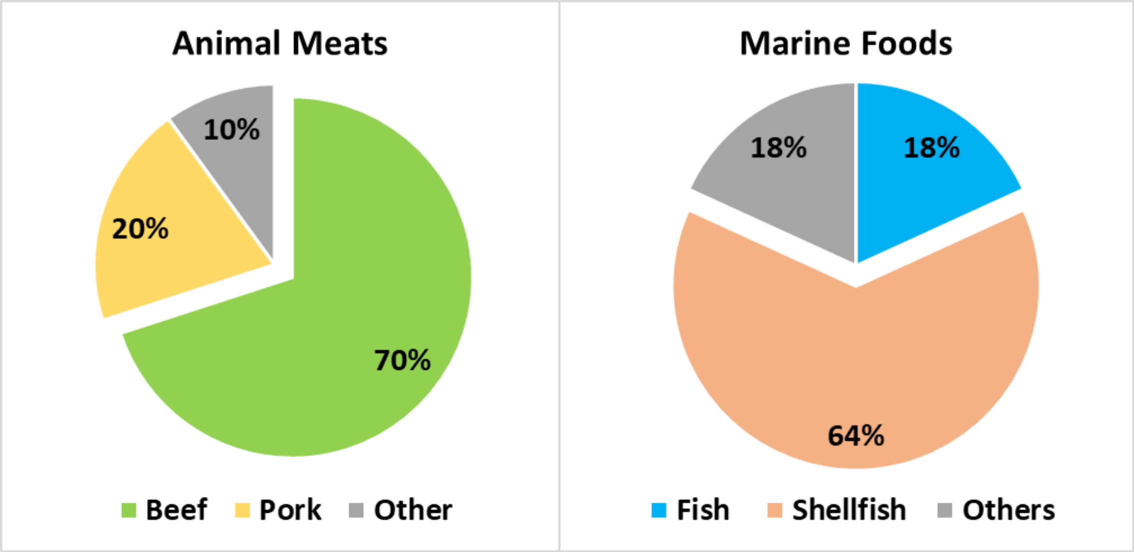

Supplementary Fig. S1. The sources of animal fat and fish oil suggested by factor analysis. The results were unconvincing, because beef was suggested to be the main source among animal meats, and shellfish was suggested as the main source among marine foods.

The top10 foods selected by independent component analysis (ICA) in each food group

1) The top10 foods in animal kinds:
1.Beef sirloin, 2. Pork bottom round, 3. Chicken breast with skin, 4. Deer meat, 5. Boiled chicken tender, 6. Frog meat, 7. Horse meat, 8. Pork cutlet, 9. Beef leg, 10. Bacon

2) The top10 foods in dairy products:
1. Lacto ice (ice cream with milk-solids content of 3% or greater), 2. Natural edam cheese, 3. Sherbet, 4. Yogurt, 5. Natural cheese cram, 6. Skimed milk, 7. Goat milk, 8. Coffee milk beverage, 9. Coffee whitener, 10. Natural cheese Camembert

3) The top10 foods in marine products:
1. Sardin can, 2. Scallop, 3. Pen shell, 4. Dried squid, 5. Boiled Octopus, 6. Sea cucumber Konowata, 7. Boiled Japanese flying squid, 8. Baked Japanese flying squid, 9. Itaya shellfish, 10. Dried Scallop

4) The top10 foods in plants:
1. Bean kind foods, 2. Carrot, 3. Boiled Bassia scoparia, 4. Bean sprouts, 5. Raw Onion Stalk, 6. Tomato, 7. Japanese red Kintoki carrot, 8. Boiled Japanese red Kintoki carrot, 9. green beans, 10. Raw Broad Beans

The top10 foods selected by factor analysis in each food group

1) The top10 foods in animal kinds:
1.Beef rib roast, 2. Beef sirloin (dairy fattening beef), 3. Beef leg without skin, 4. Raw beef tongue, 5. Cow rump, 6. Pork cartilage, 7. Pork cutlet, 8. Raw pork bottom round, 9. Bee larva can, 10. Beef sirloin (Hybrid beef)

2) The top10 foods in dairy products:
1. Breast milk, 2. Cow milk, 3. Ice-cream, 4. Lacto ice, 5. Infant milk powder, 6. Goat milk, 7. Natural goat cheese, 8. Natural edam cheese, 9. Yogurt beverage, 10. Milk powder

3) The top10 foods in marine products:
1. Boiled scallop can, 2. Boiled Asiatic hard clam in Tsukudani style, 3. Boiled Manila clam in Tsukudani style, 4. Manila clam can, 5. Raw Korean mussel, 6. Half-crenated ark can, 7. Boiled Mysidacea in Tsukudani style, 8. Sea urchins, 9. Boiled Ammodytes in Tsukudani style, 10. Gobioidei, 11. Sinonovacula, 12. Boiled oyster, 13. baked Asiatic hard clam
(The correlation coefficient values of 4~8 are the same, and the correlation coefficient values of 9~13 are the same. Tsukudani is a kind of preservable foods boiled down in soy sauce.)

4) The top10 foods in plants:
1. bean kind foods, 2. Carrot, 3. Boiled Bassia scoparia, 4. Onion Stalk, 5. Bean sprouts, 6. Japanese red Kintoki carrot, 7. Boiled Japanese red Kintoki carrot, 8. Japanese red Kintoki carrot with skin, 9. Boiled Japanese red Kintoki carrot with skin, 10. Tomato

References

1. Shrestha, R. *et al.* Change in plasma total, esterified and non-esterified capric acid concentration during a short-term oral administration of synthetic tricaprin in dogs. Anal.

Sci., **33**, 1297-1303, https://doi.org/10.2116/analsci.33.1297 (2017).

2. Shrestha, R. *et al*. Plasma capric acid concentration in healthy subjects determined by high-performance liquid chromatography. Ann. Clin. Biochem., **52**, 588-596. https://doi.org/10.1177/0004563215569081 (2015).
3. Shrestha, R. *et al*. Microwave-assisted derivatization of fatty acids for its measurement in milk using high-performance liquid chromatography. Anal. Sci., **34**, 575–582, https://doi.org/10.2116/analsci.17P557 (2018).
4. Chen, Z. *et al*. Determination of Total, Free, and Esterified Short-Chain Fatty Acid in Human Serum by LC-MS/MS, Ann. Clin. Biochem., **56**, 190-197. https://doi.org/10.1177/0004563218801393 (2019).
5. Miura, Y. *et al*. Absolute quantification of cholesteryl esters using liquid chromatography-tandem mass spectrometry uncovers novel diagnostic potential of urinary sediment, Steroids, **123**, 43-49. https://doi.org/10.1016/j.steroids.2017.05.003 (2017).
6. Miura, Y, *et al*. Synthesis of (2β,3α,6-$^2H_3$cholesteryl linoleate and cholesteryl oleate as internal standards for mass spectrometry. Steroids. **107**,1-9. https://doi.org/10.1016/j.steroids.2015.12.004 (2016).
